# Whole-Body MRI Versus ^18^F-FDG-PET/CT for the Detection of Nodal and Distant Metastases in Pediatric Patients with Solid Tumors

**DOI:** 10.3390/cancers18172749

**Published:** 2026-08-24

**Authors:** André Lollert, Maximilian Wittmann, Fabio Souschek, Markus Schepers, Matthias Miederer, Mathias Schreckenberger, Alexandra Russo, Tobias Bäuerle, Gundula Staatz

**Affiliations:** 1 Department of Diagnostic and Interventional Radiology, Section of Pediatric Radiology, University Medical Center of the Johannes Gutenberg University, 55131 Mainz, Germanygundula.staatz@unimedizin-mainz.de (G.S.); 2 Department of Diagnostic and Interventional Radiology, University Hospital Bonn, 52127 Bonn, Germany; 3 Institute of Medical Biostatistics, Epidemiology and Informatics, University Medical Center of the Johannes Gutenberg University, 55131 Mainz, Germany; 4 Department of Nuclear Medicine, University Medical Center of the Johannes Gutenberg University, 55131 Mainz, Germany; matthias.miederer@nct-dresden.de (M.M.);; 5 Department of Translational Imaging in Oncology, National Center for Tumor Diseases (NCT), NCT/UCC Dresden, a Partnership Between DKFZ (German Cancer Research Center), Faculty of Medicine, University Hospital Carl Gustav Carus, TUD (Dresden University of Technology), and Helmholtz-Zentrum Dresden-Rossendorf (HZDR), 01307 Dresden, Germany; 6 Department of Pediatrics, University Medical Center of the Johannes Gutenberg University, 55131 Mainz, Germany; 7 Department of Diagnostic and Interventional Radiology, University Medical Center of the Johannes Gutenberg University, 55131 Mainz, Germany; tobias.baeuerle@unimedizin-mainz.de

**Keywords:** whole-body MRI, ^18^F-FDG-PET/CT, pediatric oncology, pediatric radiology, staging

## Abstract

**Background/Objectives**: Pediatric patients with solid tumors (non-lymphoma) require adequate staging prior to and during therapy. ^18^F-FDG-PET/CT is commonly used for the detection of tumorous lesions, with the drawback of exposure to ionizing radiation. Whole-body MRI (WB-MRI) can be considered as a complementary method according to clinical guidelines. The main objective of this study was to assess the diagnostic performance of the aforementioned methods for the detection of metastases, both separately and in combination. **Methods**: We retrospectively assessed sensitivity and specificity of ^18^F-FDG-PET/CT, WB-MRI, and the combination of both methods for the detection of lymph node and distant metastases in pediatric patients with solid tumors. **Results**: A total of 21 patients who underwent both ^18^F-FDG-PET/CT and WB-MRI were included in this study. We found 109 confirmed metastatic lesions in 1322 body regions. Sensitivity and specificity for the whole collective were 63.4%/99.3% for ^18^F-FDG-PET/CT, 91.3%/98.6% for WB-MRI, and 100%/98.2% for the combination of both methods. In 11 therapy-naïve patients, both ^18^F-FDG-PET/CT and WB-MRI exhibited a sensitivity of 84.7%, while specificity was 98.8% for ^18^F-FDG-PET/CT and 98.5% for WB-MRI. The combination of both methods led to a significant increase in sensitivity to 100% in both therapy-naïve patients and patients under chemotherapy (both *p* < 0.001), while specificity remained high (97.7%/98.7%). **Conclusions**: ^18^F-FDG-PET/CT in combination with WB-MRI showed a higher sensitivity for the detection of metastases in solid malignant pediatric tumors than either of the methods alone. Thus, the combination allows for the most accurate staging.

## Simple Summary

When cancer is diagnosed in a child, it is essential to determine whether it has spread to other parts of the body, because this influences treatment and the chances of recovery. Positron-emission tomography combined with computed tomography is a common diagnostic test that is used for this purpose. However, it involves radiation, which is a particular concern in children. Whole-body magnetic resonance imaging is a radiation-free alternative that may improve the detection of cancer spread. In this study, we compared these two imaging methods in children with solid tumors. We found that both methods performed similarly before treatment initiation. When both imaging techniques were used in combination, all confirmed metastatic lesions were detected with high overall accuracy. These findings suggest that combining the two methods could improve cancer staging and support more informed treatment decisions for children with solid tumors.

## 1. Introduction

Among all cancer diagnoses in children, age-standardized incidence rates (ASRs) are highest for leukemias (55.4 per million in Germany), tumors of the central nervous system (43.1), and lymphomas (19.0). Pediatric solid tumors occur less frequently, for example, with ASRs of 12.7 for neuroblastoma, 9.2 for soft tissue sarcomas, and 6.5 for other bone tumors [1]. Still, accurate staging is essential for the management of pediatric patients with solid tumors, as the presence of nodal or distant metastases influences risk stratification, therapeutic decision making, and prognosis [2,3]. In many childhood malignancies, metastatic spread at diagnosis is associated with markedly poorer outcomes and often necessitates intensified multimodal treatment [4]. Consequently, imaging techniques that reliably detect metastatic disease while minimizing side effects to the patient are of central importance in pediatric oncology [5].

Hybrid imaging with ^18^F-fluorodeoxyglucose positron emission tomography/computed tomography (^18^F-FDG-PET/CT) has become an established modality for oncologic staging, including in pediatric patients, especially in the case of malignant lymphomas [6]. By combining metabolic information from PET with anatomical correlation from CT, ^18^F-FDG-PET/CT enables sensitive detection of metabolically active tumorous lesions. Thus, it can be used for the detection of distant metastases in solid tumors, primarily sarcomas, certain neuroblastomas, and, occasionally, germ cell tumors [6]. However, the use of PET/CT in children raises concerns related to cumulative ionizing radiation exposure [7,8]. Novel hybrid techniques such as PET/MRI lower the radiation dose by omitting the CT part, but are not widely available even among large academic imaging centers [9]. Additionally, the method may demonstrate limited sensitivity in pediatric tumors with low FDG avidity, and detection can vary depending on tumor biology and treatment status [10].

Whole-body magnetic resonance imaging (WB-MRI) has emerged as a radiation-free alternative for the assessment of malignant disease in several pediatric tumors [11,12,13]. Additionally, the inclusion of WB-MRI into the imaging protocol has already shown a potential to improve long-term survival in pediatric patients with Ewing sarcoma [14]. Despite the aforementioned advantages, WB-MRI has not become a first-line imaging modality in clinical guidelines yet, with exceptions for specific diseases or indications (e.g., B-cell non-Hodgkin lymphoma or screening in patients with a tumor predisposition syndrome) [15,16]. In most cases, it is recommended as a method to be considered in addition to standard imaging modalities. However, evidence for an increase in sensitivity if WB-MRI is performed in addition to PET/CT is still lacking, as most of the published studies assessed a comparison of both methods with each other, but not to their combination [11,17,18].

Therefore, the aim of this study was to compare the diagnostic performance of ^18^F-FDG-PET/CT and WB-MRI alone as well as the combination of both methods for the detection of lymph node and distant metastases in pediatric patients with solid tumors (non-lymphoma).

## 2. Materials and Methods

### 2.1. Patients

According to local regulations, approval by the local Independent Ethics Committee was not necessary due to the retrospective study design. We evaluated imaging data of all pediatric patients (i.e., below 18 years of age) who underwent at least one ^18^F-FDG-PET/CT and WB-MRI examination for the staging of a solid tumor between 2012 and 2022. Exclusion criteria were a diagnosis of lymphoma, a time interval of more than 90 days between the two examinations, non-completed examinations due to patient incompliance, and no detectable lesions on both modalities. The latter exclusion was performed because the cohort would otherwise have been enriched with true-negative cases, potentially resulting in an artificially high specificity. In addition, the follow-up imaging strategy differs substantially for patients without metastatic disease at initial staging, which complicates the assessment of newly developing lesions during follow-up. Furthermore, we divided the collective into subgroups of therapy-naïve patients (subgroup 1) and patients under chemotherapy (subgroup 2). To account for the high heterogeneity of the included tumors, we performed an additional descriptive subgroup analysis of patients with small round blue cell tumors (SRBCTs, i.e., Ewing sarcoma, rhabdomyosarcoma, and neuroblastoma), as these were the most frequent tumor entities with similar pathohistological characteristics in our cohort.

### 2.2. ^18^F-FDG-PET/CT

The legal guardians of all patients provided written informed consent for the clinically indicated ^18^F-FDG-PET/CT examinations of the whole body, which were acquired on a Gemini^®^ TF 16 PET/CT system (Philips, Best, The Netherlands) 60 min after the application of ^18^F-FDG (2.5–3 MBq/kg body weight). A fasting period of more than six hours before application was mandatory for all patients. Low-dose CT without contrast media was performed for attenuation correction. We applied sedation in small children depending on patient compliance.

### 2.3. Whole-Body MRI

The legal guardians of all patients provided written informed consent for the clinically indicated WB-MRI examinations. We applied sedation in small children depending on patient compliance. Examinations were performed on a 1.5 Tesla scanner (Magnetom Avanto^®^, Siemens Healthineers, Erlangen, Germany), or two different 3 T scanners (Magnetom Skyra^®^ or Magnetom Vida^®^, Siemens Healthineers, Erlangen, Germany). The standard protocol consisted of a whole-body T2-weighted coronal turbo-inversion recovery-magnitude (TIRM) sequence, a sagittal T2-weighted TIRM sequence of the whole spine, a transverse T2-weighted TIRM sequence with radial k-space sampling (BLADE) covering the neck and thorax, and a transverse breath-triggered T2-weighted turbo spin echo sequence with fat saturation for the abdomen. Technical details of the sequences are summarized in Table 1. Additional sequences were performed as clinically determined by the radiologist.

### 2.4. Lesion Analysis

We assessed lesions based on anatomic location adapted from Klenk et al. [18]: Nodal regions consisted of Waldeyer’s ring, cervical region, mediastinum, pulmonary hilum, supraclavicular region, axillary region, spleen, abdominal and para-aortic region, mesenteric region, retrocrural region, retroperitoneal region, pelvic region, omental region and peritoneum, and inguinal region. Extra-nodal regions included the lung, pleura, breast, liver, pancreas, adrenal gland, kidney, small intestines, large intestines, gallbladder, urinary bladder, uterus, ovaries, testes, skull, cervical spine, thoracic spine, lumbar spine, sacrum, coccyx, ribs, clavicle, scapula, sternum, humerus, forearm and hand, pelvic bone, femur, and lower leg and foot. In bilateral regions, the right and left sides were assessed independently.

Lesions were classified as suspicious or non-suspicious based on clinical reports from our radiology information system. Lesions described as “potentially suspicious” were considered suspicious in all analyses. Ambiguous cases were retrospectively reviewed by a pediatric radiologist with more than 10 years of expertise in pediatric MRI reading (A.L.) or a nuclear medicine specialist with more than 15 years of expertise in pediatric PET/CT reading (M.M.). The reference standard for every lesion was established by histology or imaging follow-up (i.e., lesion progression without therapy or lesion regression under chemotherapy). Lesions without a reference standard due to missing histology or follow-up were excluded from further analyses. The primary tumor was not included in the analyses, and its region was counted positive only if additional metastatic lesions were present in the same region.

### 2.5. Statistics

Descriptive statistics were used to characterize the patient collective. For the primary analysis, the diagnostic performance (sensitivity and specificity) of ^18^F-FDG-PET/CT, WB-MRI, and their combination was evaluated. The combination was considered positive if either modality yielded a positive finding.

To account for the intra-patient correlation (clustering) of multiple lesions within the same individual, generalized estimating equations (GEEs) with an exchangeable correlation structure and a binomial distribution (logit link function) were employed. Separate GEE models were constructed to estimate sensitivity (restricted to truly positive lesions) and specificity (restricted to truly negative lesions). Pairwise comparisons between modalities were performed using the emmeans package with Tukey adjustment for multiple comparisons.

All analyses were performed for the total study population and for two subgroups: therapy-naïve patients and patients undergoing chemotherapy. Diagnostic accuracy was further characterized by reporting raw counts of true positives, false positives, true negatives, and false negatives.

Statistical analyses were performed using R (version 4.5.2; R Foundation for Statistical Computing, Vienna, Austria) using the geepack, tidyverse, and emmeans packages, as well as SPSS Statistics (version 29.0; IBM, Armonk, NY, USA). A *p*-value < 0.05 was considered statistically significant. As this was an exploratory study, no further adjustments for multiple testing were made.

## 3. Results

**Figure 1:**
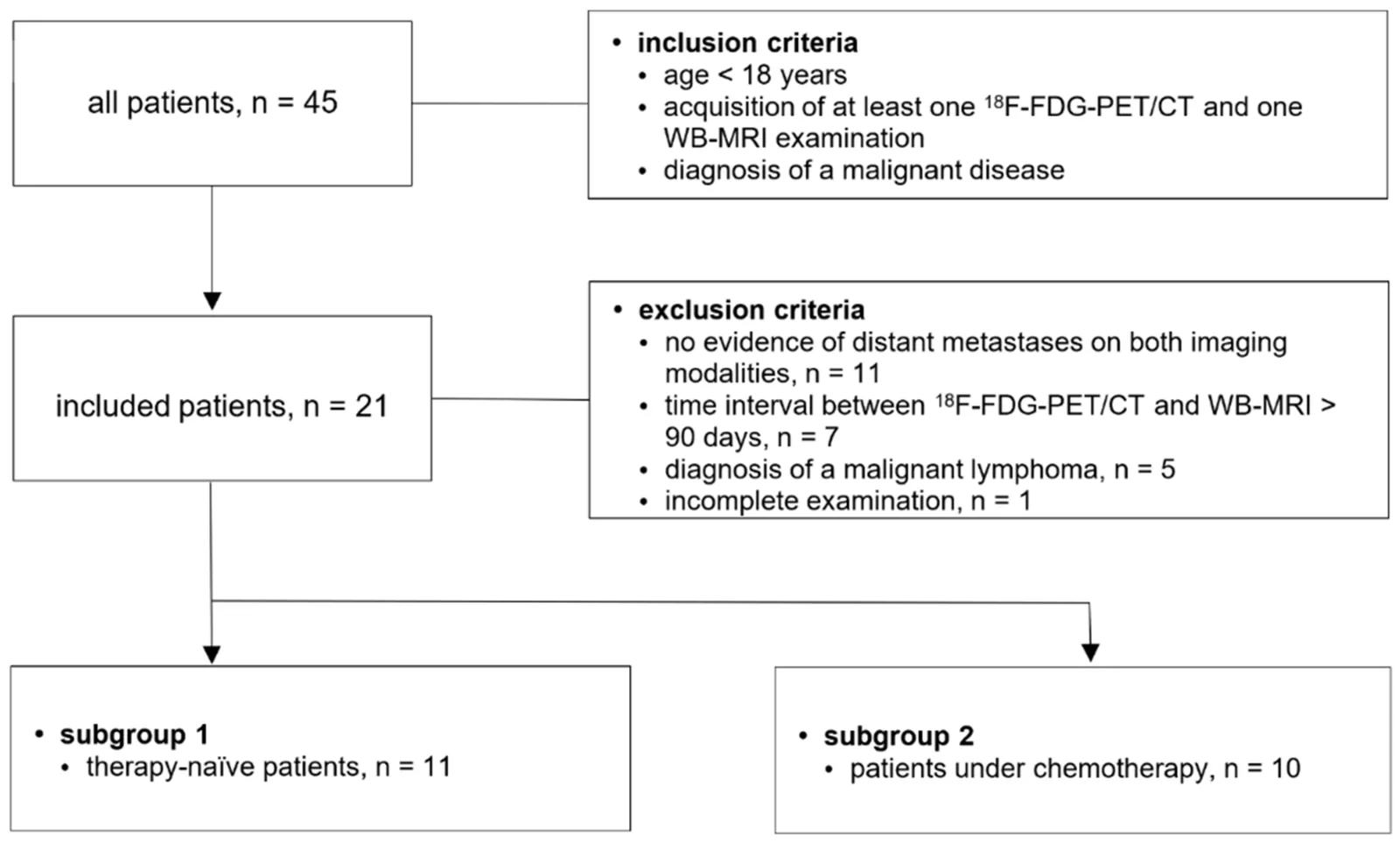
Flow chart of the included and excluded examinations.

In our database, we found 45 consecutive pediatric patients with a malignant disease who underwent both ^18^F-FDG-PET/CT and WB-MRI for staging. Patients were excluded if they had no evidence of distant metastases (n = 11), the time interval between ^18^F-FDG-PET/CT and WB-MRI was longer than 90 days (n = 7), due to a diagnosis of lymphoma (n = 5), or the examinations were not successfully completed due to patient incompliance (n = 1). Thus, our study cohort consisted of 21 patients (Table 2). A flow chart is depicted in Figure 1.

The mean time interval (and standard deviation) between the ^18^F-FDG-PET/CT and WB-MRI examinations was 10 ± 8 days (range 1 to 35 days). In most of the cases (19/21), WB-MRI was performed prior to ^18^F-FDG-PET/CT. Furthermore, 11 of 21 patients were therapy-naïve; in 10 patients, imaging was performed under chemotherapy. In the latter group, the mean time interval between therapy initiation and the WB-MRI examination was 48 ± 69 days. For the ^18^F-FDG-PET/CT examination, this time interval was 51 ± 69 days. Periprocedural sedation was required in 9/21 cases (43%) for the WB-MRI and in 2/21 cases (10%) for the ^18^F-FDG-PET/CT examination.

### 3.1. Results of Lesion Analyses

In a total of 1322 analyzed body regions in 21 patients, 138 suspicious (of these: 130 suspicious, 8 “potentially” suspicious) lesions were detected. Seven lesions were excluded, as no reference standard could be determined. Of the remaining 131 lesions, 109 were confirmed as metastatic, while we classified 22 lesions as non-metastatic. The reference standard was confirmed by histology in 7 cases and based on the follow-up in 124 cases.

Table 3 summarizes raw diagnostic performance counts for the whole collective and both subgroups, depending on the modality. In Table 4, corresponding sensitivity and specificity analyses are shown.

In the whole collective, WB-MRI demonstrated a significantly higher overall sensitivity compared with ^18^F-FDG-PET/CT (91.3% vs. 63.4%, *p* < 0.001). The observed sensitivity of the combined approach (100.0%) was significantly higher than that of either modality alone (*p* < 0.001). Specificity remained high across all approaches, ranging from 98.2% to 99.3%. The diagnostic performance varied substantially depending on the treatment status: In subgroup 1 (therapy-naïve patients), sensitivity was comparable between ^18^F-FDG-PET/CT and WB-MRI (84.7% for both; *p* = 1.00). Specificity was also similar (98.8% vs. 98.5%; *p* = 0.95). In subgroup 2 (patients under chemotherapy), the sensitivity of ^18^F-FDG-PET/CT decreased significantly to 53.2% (95% confidence interval [CI]: 39.5–66.5%), while WB-MRI maintained a high sensitivity of 98.0% (95% CI: 88.7–99.7%). This difference was statistically significant (*p* = 0.006). Conversely, ^18^F-FDG-PET/CT showed a slightly but significantly higher specificity (99.8%) compared with WB-MRI (98.7%; *p* = 0.01). Of note, the combination of both imaging modalities achieved a sensitivity of 100% in both subgroups. The specificity for this combined approach remained excellent at 97.7% in subgroup 1 and 98.7% in subgroup 2. Pairwise comparisons confirmed that the combination was significantly more sensitive than either modality alone across the entire analysis population (*p* < 0.001).

The results of the additional subgroup analyses, including only patients with SRBCTs, are presented in Supplementary Tables S1 and S2. Interestingly, in this subgroup, ^18^F-FDG-PET/CT detected only one additional confirmed metastatic lesion that was missed by WB-MRI.

### 3.2. Description of False Negative Lesions

**Figure 2:**
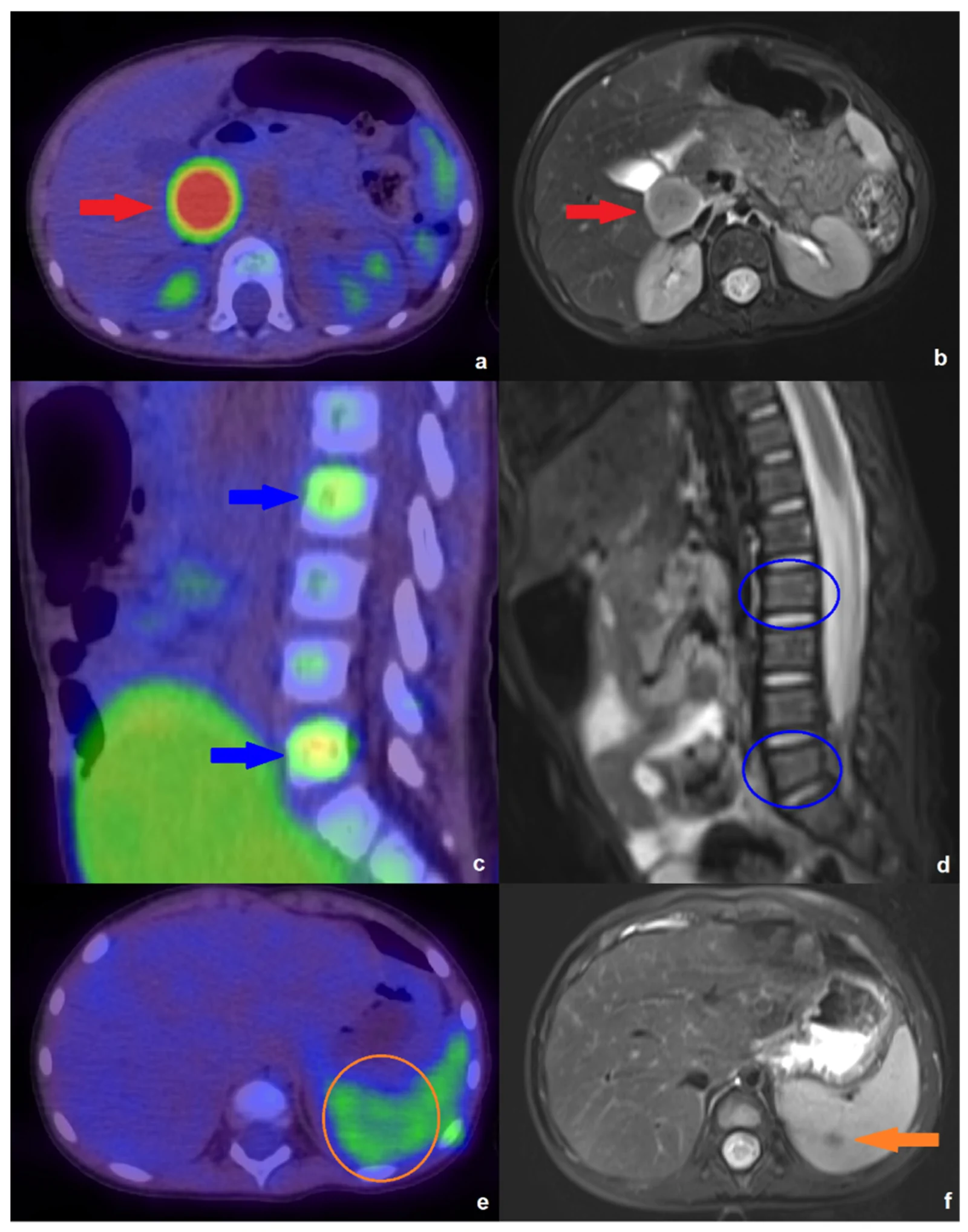
Fused PET/CT with FDG uptake being color-coded from low (blue) via green, yellow and orange to red (very high), (**a**) and MR (transverse T2-weighted TSE sequence with fat saturation), (**b**) images demonstrating an FDG-avid lesion in the right adrenal gland (red arrows). In the same patient, metastatic lesions in the lumbar spine were detected only by an increased FDG uptake (blue arrows, (**c**)) with no visible signal alterations in a corresponding TIRM MRI sequence (blue circles, (**d**)). In contrast, splenic involvement was not detected by PET/CT (orange circle, (**e**)), but was visible in the MRI (orange arrow, (**f**)).

In the subgroup of therapy-naïve patients, WB-MRI failed to identify a total of seven lesions that were detected by ^18^F-FDG-PET/CT. All of these lesions occurred in a 17-month-old girl with a mass in the right adrenal gland in addition to several osteolytic lesions. Therefore, the clinical suspicion was either metastatic neuroblastoma or multifocal Langerhans cell histiocytosis. While the adrenal gland tumor was visible on both WB-MRI and ^18^F-FDG-PET/CT, some of the osseous lesions were detected only by ^18^F-FDG-PET/CT. In contrast, two splenic lesions were detected only by WB-MRI (Figure 2). The diagnosis of Langerhans cell histiocytosis was confirmed by histology.

**Figure 3:**
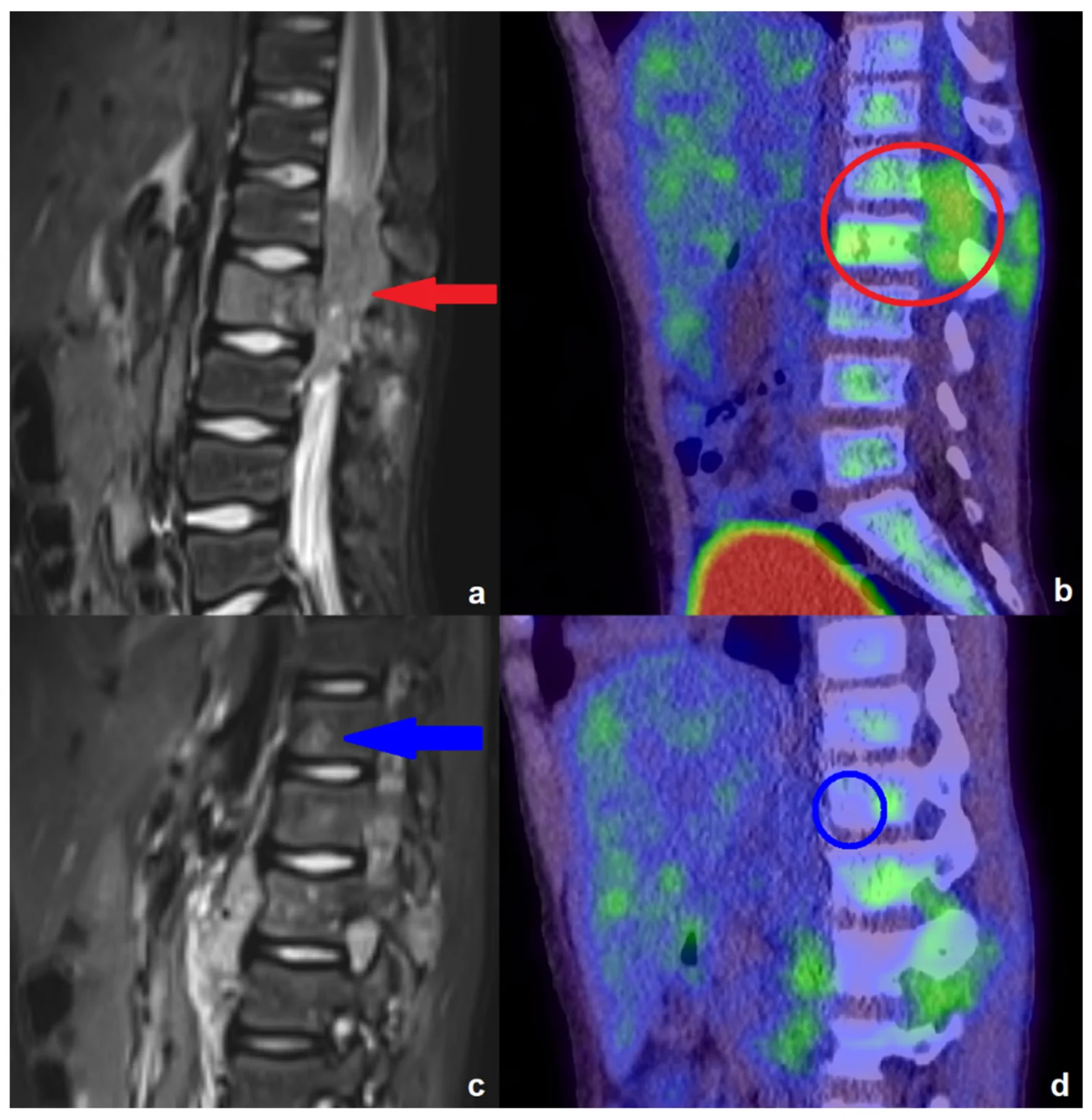
MR image (sagittal TIRM sequence) demonstrating a Ewing sarcoma originating from the lumbar spine with intraosseous and intraspinal growth (red arrow, (**a**)) with a low FDG uptake on the corresponding PET/CT image (color-coded as in Figure 2, red circle, (**b**)). A metastatic lesion in the lower thoracic spine (confirmed by complete regression under chemotherapy during follow-up) was only visible on the MRI (blue arrow, (**c**)) but not on the PET/CT scan (blue circle, (**d**)) due to missing FDG uptake.

Some tumors demonstrated low FDG avidity, which led to false negative results on ^18^F-FDG-PET/CT. For example, a Ewing sarcoma of the lumbar spine with intraspinal tumor growth in a 9-year-old patient exhibited low but detectable FDG avidity. However, osseous metastases (confirmed by regression under chemotherapy during follow-up) in the upper and lower thoracic spine were visible only on WB-MRI (Figure 3).

Other lesions missed by ^18^F-FDG-PET/CT were located in the brain, liver, and other bones. Particularly, brain metastases altered treatment, as whole-brain radiotherapy was indicated, for example in a 17-year-old boy with a chorion carcinoma of the upper abdomen. One lesion in the left lung was initially classified nonspecific on ^18^F-FDG-PET/CT, but exhibited increased FDG uptake in the follow-up.

False negative lesions on ^18^F-FDG-PET/CT in the subgroup under chemotherapy mainly occurred when FDG uptake in metastatic lesions decreased, while residual signal alterations remained visible on WB-MRI.

### 3.3. Description of False Positive Lesions

All lesions that were classified as false positives on ^18^F-FDG-PET/CT while being determined negative on WB-MRI occurred in the same patient: an 11-year-old boy with Ewing sarcoma of the femur. Due to the moderate FDG uptake of the primary tumor, several lymph nodes as well as osseous areas with slightly increased uptake were incorrectly classified as suspicious.

**Figure 4:**
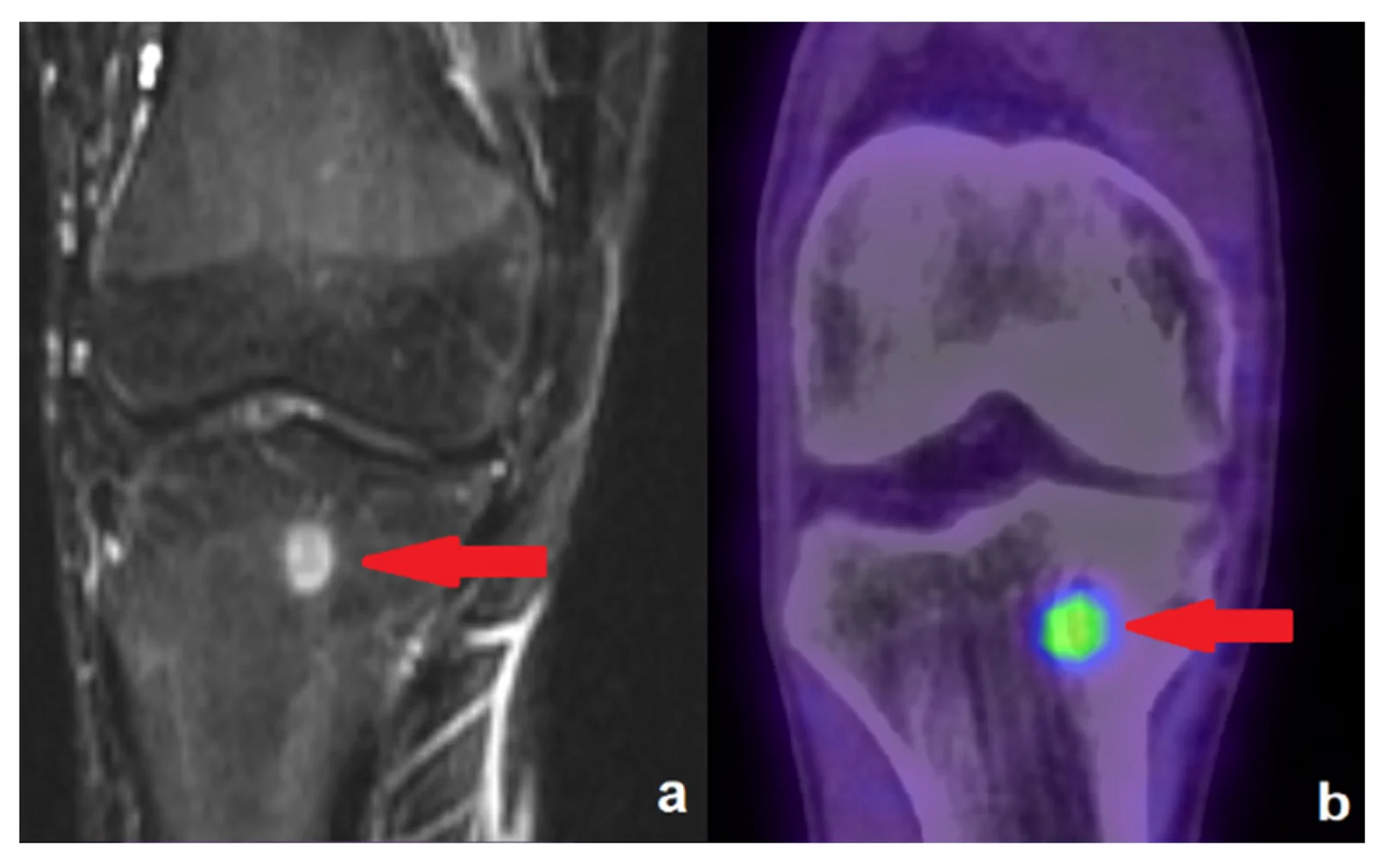
MR image (coronal T2-weighted TIRM sequence) demonstrating a focal hyperintense lesion in the proximal right tibia (red arrow, (**a**)) with an increased FDG uptake on the corresponding PET/CT image (color-coded as in Figure 2, (**b**)).

In three therapy-naïve patients, lesions were incorrectly classified as metastases on both modalities: In a 17-year-old boy with a chorion carcinoma of the upper abdomen (the same patient as presented in Section 3.2), a focal hyperintense lesion with increased FDG uptake was detected in the right proximal tibia (Figure 4).

Biopsy revealed bone tissue with stromal fibrosis and no signs of malignant infiltration. Therefore, the concordant imaging findings altered the diagnostic process, leading to an invasive biopsy. Because the biopsy was negative, otherwise-indicated local radiotherapy was not performed. In two 15-year-old female patients with Ewing sarcomas, lesions in an axillary lymph node and in the left ovary were classified as suspicious based on MR characteristics and FDG uptake. However, histology did not show neoplastic infiltration. Notably, histopathological evaluation was performed after the initiation of chemotherapy in both cases. This also applies to a patient with neuroblastoma who was imaged under chemotherapy; histologic evaluation of a suspicious bone lesion was negative.

False positive lesions on WB-MRI that were correctly classified as negative on ^18^F-FDG-PET/CT were osseous (n = 8), nodal (n = 4), or located in the lung (n = 1).

## 4. Discussion

In this study, we compared the diagnostic performance of ^18^F-FDG-PET/CT, WB-MRI, and their combination for detecting lymph node and distant metastases in pediatric patients with solid tumors. Three main findings emerged. First, ^18^F-FDG-PET/CT and WB-MRI showed comparable diagnostic performance in therapy-naïve patients. Second, in our exploratory cohort, WB-MRI showed higher observed sensitivity than ^18^F-FDG-PET/CT in patients undergoing chemotherapy. Third, combining both modalities yielded the highest diagnostic accuracy, achieving a very high sensitivity with only a minimal reduction in specificity.

The comparable performance of ^18^F-FDG-PET/CT and WB-MRI in therapy-naïve patients aligns with previous studies that reported similar sensitivities of both modalities for the initial staging in pediatric malignancies [11,18]. Of note, only two patients in the latter study [18] had solid tumors, and most data were based on patients with lymphoma. In contrast, a recent study focusing on bone and soft-tissue sarcomas exhibited a higher sensitivity for conventional WB-MRI (42.6%) compared with ^18^F-FDG-PET/CT (30.2%) [19]. The relatively low sensitivities observed in that study may be explained by methodological differences, particularly the use of lesion-based rather than region-based reference standards as determined in our study or in the studies by Kumar et al. [11] and Klenk et al. [18].

In contrast to the aforementioned studies, our results are based on a heterogeneous group of different tumors, with sarcoma being the most frequent entity. This heterogeneity may influence the overall analysis, as some of the included entities, particularly neuroblastoma, have a higher propensity for metastatic disease [20]. In addition, the 10 tumor entities included in our cohort comprise biologically heterogeneous pediatric malignancies with distinct clinical characteristics and FDG uptake patterns as well as entity-specific treatment responses [21]. Thus, the reported sensitivity and specificity values may primarily reflect the composition of this particular cohort rather than a true effect that is generalizable to all pediatric solid tumors. In the exploratory SRBCT subgroup, WB-MRI showed higher observed sensitivity than ^18^F-FDG-PET/CT, while the latter modality detected only one additional metastatic lesion not identified by WB-MRI. These findings should be interpreted cautiously given the small subgroup size.

^18^F-FDG-PET/CT benefits from its ability to detect metabolically active lesions, which is particularly advantageous in untreated tumors with high glucose metabolism [22]. However, in this study, ^18^F-FDG-PET/CT failed to detect metastatic lesions in different anatomical regions, including the brain and spleen for methodological reasons, i.e., high inherent glucose metabolism [23], and bone, particularly in tumors with low FDG avidity such as some Ewing sarcomas. In contrast, false negative findings on WB-MRI were rare and occurred in only a single therapy-naïve patient. This highlights that WB-MRI is highly sensitive to bone marrow and soft-tissue involvement [24]; however, it may still fail to detect certain lesions, particularly small or early osseous manifestations.

False positive lesions on WB-MRI were predominantly observed in bone and lymph nodes, in line with the data presented by Klenk et al. [18]—22 bone lesions and 4 lymph nodes in their study. Of note, only one bone lesion in our study that appeared suspicious on both imaging modalities was ultimately proven benign by biopsy. In the other cases, negative histopathological results may have been influenced by ongoing chemotherapy at the time of the biopsy.

The significantly higher sensitivity of WB-MRI during chemotherapy can be explained by treatment-induced changes in tumor biology. Chemotherapy often reduces metabolic activity before substantial morphological regression occurs, potentially leading to false-negative findings on ^18^F-FDG-PET/CT [25]. Still, decreased FDG uptake is widely used for therapy response assessment [26], raising the question of how to define true remission—whether based on complete metabolic response or complete regression of imaging abnormalities on MRI [27].

The most important finding of our study is the substantial improvement in diagnostic performance achieved by combining ^18^F-FDG-PET/CT and WB-MRI, which corresponds to the adult literature, for example for the staging of thyroid carcinoma [28]. While the combination of WB-MRI and ^18^F-FDG-PET/CT yielded a sensitivity of 100% in our cohort, the lower bounds of the 95% confidence intervals (e.g., 92.0% in the therapy-naïve group) reflect the statistical uncertainty inherent in our small, retrospective sample. A single additional false-negative result would have noticeably altered this percentage. However, false-negative lesions are difficult to assess on follow-up imaging, as it is unclear whether a new lesion was already undetectably present on the initial examination or developed later. Nevertheless, when two imaging modalities are combined in a parallel testing strategy in which a lesion is considered positive if detected by either modality, an increase in sensitivity is partly expected by definition. Therefore, these results should be interpreted as highly promising rather than definitive and warrant validation in larger, prospective cohorts. Previous studies in pediatrics have primarily focused on direct comparisons between those modalities rather than their combined use [11,18,19]. From a clinical perspective, maximizing sensitivity is essential in pediatric oncology staging, as missed metastatic lesions may result in undertreatment and poorer outcomes. Our findings therefore support a multimodal imaging approach. Similarly, prior work has demonstrated excellent sensitivity for detecting bone marrow metastases when performing a combination of ^18^F-FDG-PET with diffusion-weighted WB-MRI on an integrated PET/MRI scanner [29].

Despite these promising results, practical considerations must be addressed. Radiation-free WB-MRI is associated with longer acquisition times and frequently requires sedation in younger children, as reflected by the higher sedation rate in our cohort. In contrast, ^18^F-FDG-PET/CT examinations are typically faster, but radiation exposure is of concern, particularly in pediatric patients. Hybrid PET/MRI systems may offer a promising solution by combining metabolic and high-contrast anatomical imaging while reducing radiation exposure, although limited availability and higher costs currently restrict widespread implementation [30]. In addition, the CT component of ^18^F-FDG-PET/CT imaging, despite being acquired with low-dose protocols, might be useful for lesion characterization, especially to differentiate possibly benign diagnoses with increased FDG uptake (e.g., non-ossifying fibroma) [31]. Furthermore, lung metastases might be more reliably detected by (low-dose) CT compared with MRI [32,33].

### Limitations

Our study has several limitations. First, its retrospective design may introduce selection bias. In addition, the study relied primarily on the original clinical reports, with retrospective reassessment performed only in selected cases, which may have introduced observer bias. However, this approach reflects real clinical decision making based on the information available at the respective time point. Furthermore, as discussed above, the cohort includes a heterogeneous group of tumor entities, which may limit generalizability. The exclusion of patients without detectable metastatic disease, although undertaken for the reasons described in Section 2.1, may have introduced spectrum bias, potentially inflating estimates of diagnostic performance. The reference standard was predominantly based on imaging follow-up rather than histopathological confirmation, which is understandable in pediatric oncology but inevitably introduces verification bias and limits confidence in the reported diagnostic accuracy measures. Although the interval between ^18^F-FDG-PET/CT and WB-MRI was limited to a maximum of 35 days, disease progression or treatment effects during this period may have influenced lesion detectability. In addition, the fact that the majority of WB-MRI examinations were performed before ^18^F-FDG-PET/CT could introduce temporal bias. Consequently, some observed differences between modalities may reflect temporal changes in disease status rather than true differences in diagnostic accuracy. Furthermore, diffusion-weighted imaging (DWI) was not included in our WB-MRI protocol, although it has shown the potential to improve sensitivity in a prior study [19]. Accordingly, the comparison may not fully reflect contemporary WB-MRI performance. However, including DWI into the standard WB-MRI protocol would cause significantly longer examination times, i.e., more than 4 min per body section [19]. Finally, while lesion-based analysis increases statistical power, it does not fully reflect patient-level clinical decision making.

To account for the aforementioned limitations, future research is needed to validate our findings, ideally through larger, prospective, multicenter studies using standardized imaging protocols including novel techniques such as DWI. For more frequently occurring tumors, such as sarcoma, these studies could be tumor-specific, whereas basket studies including different tumor types may be more suitable for those with very low incidence rates. Finally, in larger cohorts, patient-level accuracy analyses may help to clarify the clinical implications of potentially increased sensitivity achieved through the combination of modalities.

## 5. Conclusions

In conclusion, ^18^F-FDG-PET/CT and WB-MRI demonstrated complementary strengths in detecting metastatic disease in pediatric solid tumors. While both modalities performed similarly in therapy-naïve patients, WB-MRI showed superior sensitivity during chemotherapy. However, this finding needs to be confirmed in prospective studies with a particular focus on the definition of residual tumor burden, given the relatively small number of patients in the subgroups in our study. Accordingly, the corresponding analyses should be interpreted as exploratory. Nevertheless, most importantly, the combination of both modalities significantly improved diagnostic accuracy and achieved a very high observed sensitivity of 100% across all subgroups, with the lower bounds of the 95% confidence intervals starting at 92%, although this estimate is subject to statistical uncertainty and is partly expected from the parallel-test definition. These findings support further prospective evaluation of WB-MRI as part of a multimodal imaging strategy and highlight the potential benefit of combined imaging approaches. Particularly, the clinically meaningful question of whether the additional lesions detected by the combined approach may alter patient staging, risk stratification, or therapeutic management needs to be addressed.

## Figures and Tables

**Table 1:** Technical data of the MRI acquisition parameters.

	MRI Scanner 1	MRI Scanner 2	MRI Scanner 3
Manufacturer	Siemens Healthineers	Siemens Healthineers	Siemens Healthineers
Model	Magnetom Avanto	Magnetom Skyra	Magnetom Vida
Field strength	1.5 Tesla	3 Tesla	3 Tesla
Coronal TIRM			
TR ^1^	6000 ms	4000 ms	4000 ms
TE ^2^	57 ms	290 ms	289 ms
TI ^3^	160 ms	200 ms	220 ms
Slice thickness	6 mm	4 mm	4 mm
Interslice gap	0.6 mm	0.4 mm	0.4 mm
Flip angle	150°	120°	120°
FoV ^4^	450 mm	450 mm	450 mm
Scan matrix	384	512	512
Sagittal TIRM			
TR ^1^	7160 ms	3500 ms	3590 ms
TE ^2^	70 ms	43 ms	43 ms
TI ^3^	170 ms	215 ms	215 ms
Slice thickness	4 mm	3 mm	3 mm
Interslice gap	0.4 mm	0.3 mm	0.3 mm
Flip angle	150°	120°	150°
FoV ^4^	400 mm	380 mm	350 mm
Scan matrix	320	320	320
Transverse BLADE			
TR ^1^	6140 ms	3790 ms	8370 ms
TE ^2^	85 ms	76 ms	76 ms
TI ^3^	140 ms	200 ms	200 ms
Slice thickness	5.5 mm	5 mm	5 mm
Interslice gap	1.325 mm	0.5 mm	0.5 mm
Flip angle	121°	120°	149°
FoV ^4^	260 mm	280 mm	280 mm
Scan matrix	384	320	320
Transverse T2 TSE fs			
TR ^1^	5000 ms	4000 ms	2460 ms
TE ^2^	69 ms	99 ms	99 ms
Slice thickness	5 mm	5 mm	5 mm
Interslice gap	0.5 mm	0.5 mm	0.5 mm
Flip angle	150°	160°	160°
FoV ^4^	300 mm	220 mm	300 mm
Scan matrix	320	384	384

^1^ TR: repetition time; ^2^ TE: echo time; ^3^ TI: inversion time; ^4^ FoV: field of view.

**Table 2:** Gender (n) and age (years) of the included patients by diagnosis.

Diagnosis	Male	Female	Age(s)
Ewing sarcoma	4	3	4, 9, 11, 12, 15, 15, 16
rhabdomyosarcoma	4	1	1, 4, 4, 8, 10
neuroblastoma	1	1	1, 8
osteosarcoma	1	-	15
sarcoma of the liver	1	-	13
atypical teratoid/rhabdoid tumor (AT/RT)	-	1	1
Langerhans cell histiocytosis (LCH)	-	1	1
carcinoma of the adrenal cortex	1	-	11
yolk sac tumor	-	1	9
chorion carcinoma	1	-	17
total	13	8	median: 9; IQR ^1^: 4–14; range: 1–17

^1^ IQR: interquartile range.

**Table 3:** Raw diagnostic performance counts by group and modality.

Group	Modality	True Positive	False Positive	False Negative	True Negative
Whole collective	^18^F-FDG-PET/CT	69	9	40	1204
	WB-MRI	100	17	9	1196
	combination	109	22	0	1191
Subgroup 1: therapy-naïve patients	^18^F-FDG-PET/CT	37	8	7	644
	WB-MRI	37	10	7	642
	combination	44	15	0	637
Subgroup 2: patients under chemotherapy	^18^F-FDG-PET/CT	32	1	33	560
	WB-MRI	63	7	2	554
	combination	65	7	0	554

**Table 4:** Results of the sensitivity and specificity analyses assorted by group and modality.

Group	Modality	Sensitivity [% (95% CI ^1^)]	Specificity [% (95% CI ^1^)]
Whole collective	^18^F-FDG-PET/CT	63.4 (41.3–81.1)	99.3 (97.9–99.8)
	WB-MRI	91.3 (80.9–96.3)	98.6 (97.4–99.3)
	combination	100.0 (96.7–100.0)	98.2 (96.8–99.0)
Subgroup 1: therapy-naïve patients	^18^F-FDG-PET/CT	84.7 (71.1–92.6)	98.8 (96.2–99.6)
	WB-MRI	84.7 (72.5–92.1)	98.5 (97.0–99.3)
	combination	100.0 (92.0–100.0)	97.7 (95.6–98.8)
Subgroup 2: patients under chemotherapy	^18^F-FDG-PET/CT	53.2 (39.5–66.5)	99.8 (98.9–100.0)
	WB-MRI	98.0 (88.7–99.7)	98.7 (96.3–99.6)
	combination	100.0 (94.5–100.0)	98.7 (96.3–99.6)

^1^ CI: confidence interval.

## Data Availability

A.L. and G.S. had full access to all the data in the study and take responsibility for the integrity of the data and the accuracy of the data analysis. The data presented in this study are available on request from the corresponding authors. The data are not publicly available due to privacy (medical data).

## Supplementary Materials

The following supporting information can be downloaded at: https://www.mdpi.com/article/10.3390/cancers18172749/s1, Table S1: Analysis of the subgroup of patients with small round blue cell tumors (SRBCT): Raw diagnostic performance counts by group and modality; Table S2: Analysis of the subgroup of patients with small round blue cell tumors (SRBCT): Results of the sensitivity and specificity analyses assorted by group and modality.

## Abbreviations

The following abbreviations are used in this manuscript:

ASRs	age-standardized incidence rates
CI	confidence interval
CT	computed tomography
DWI	diffusion-weighted imaging
^18^F-FDG	^18^F-fluorodeoxyglucose
FoV	Field of view
GEEs	generalized estimating equations
PET	positron emission tomography
TE	echo time
TI	inversion time
TIRM	turbo-inversion recovery-magnitude
TR	repetition time
SRBCTs	small round blue cell tumors
WB-MRI	whole-body magnetic resonance imaging

## References

[1] Erdmann F., Wellbrock M., Trübenbach C., Grabow D., Schrappe M., Kaatsch P., Spix C., Schüz J., Ronckers C.M. (2026). Incidence patterns and temporal trends of childhood cancer in Germany, 1980-2019: Forty years of childhood cancer registration in Germany. Int. J. Cancer.

[2] de Faria L.L., Ponich Clementino C., Véras F., Khalil D.D.C., Otto D.Y., Oranges Filho M., Suzuki L., Bedoya M.A. (2024). Staging and Restaging Pediatric Abdominal and Pelvic Tumors: A Practical Guide. Radiographics.

[3] Voss S.D. (2018). Staging and following common pediatric malignancies: MRI versus CT versus functional imaging. Pediatr. Radiol..

[4] Weiss A.R., Ferrari A., Mascarenhas L., Bisogno G. (2025). Current Approaches to the Treatment of Pediatric Soft Tissue Sarcomas: Rhabdomyosarcoma and Nonrhabdomyosarcoma Soft Tissue Sarcomas. Hematol. Oncol. Clin. N. Am..

[5] van Ewijk R., Schoot R.A., Sparber-Sauer M., Ter Horst S.A.J., Jehanno N., Borgwardt L., de Keizer B., Merks J.H.M., de Luca A., McHugh K. (2021). European guideline for imaging in paediatric and adolescent rhabdomyosarcoma - joint statement by the European Paediatric Soft Tissue Sarcoma Study Group, the Cooperative Weichteilsarkom Studiengruppe and the Oncology Task Force of the European Society of Paediatric Radiology. Pediatr. Radiol..

[6] Stauss J., Franzius C., Pfluger T., Juergens K.U., Biassoni L., Begent J., Kluge R., Amthauer H., Voelker T., Højgaard L. (2008). Guidelines for 18F-FDG PET and PET-CT imaging in paediatric oncology. Eur. J. Nucl. Med. Mol. Imaging.

[7] Kim Y.Y., Shin H.J., Kim M.J., Lee M.J. (2016). Comparison of effective radiation doses from X-ray, CT, and PET/CT in pediatric patients with neuroblastoma using a dose monitoring program. Diagn. Interv. Radiol..

[8] Pearce M.S., Salotti J.A., Little M.P., McHugh K., Lee C., Kim K.P., Howe N.L., Ronckers C.M., Rajaraman P., Craft A.W. (2012). Radiation exposure from CT scans in childhood and subsequent risk of leukaemia and brain tumours: A retrospective cohort study. Lancet.

[9] Gatidis S., Ponisio M.R., Borgwardt L., Cain T.M., Francis P., Hamdi M., Johnson G., Kurch L., Laforest R., Lim R. (2025). Pediatric PET/MRI: Imaging Techniques, Indications, and Clinical Implementation. Radiographics.

[10] Franzius C. (2010). FDG-PET/CT in pediatric solid tumors. Q. J. Nucl. Med. Mol. Imaging.

[11] Kumar J., Seith A., Kumar A., Sharma R., Bakhshi S., Kumar R., Agarwala S. (2008). Whole-body MR imaging with the use of parallel imaging for detection of skeletal metastases in pediatric patients with small-cell neoplasms: Comparison with skeletal scintigraphy and FDG PET/CT. Pediatr. Radiol..

[12] Smets A.M., Deurloo E.E., Slager T.J.E., Stoker J., Bipat S. (2018). Whole-body magnetic resonance imaging for detection of skeletal metastases in children and young people with primary solid tumors - systematic review. Pediatr. Radiol..

[13] Valduga S.G., Forte G.C., Paganin R.P., Abreu D.G., Medeiros T.M., Irion K., Hochhegger B., Mattiello R. (2021). Whole-body magnetic resonance imaging for the diagnosis of metastasis in children and adolescents: A systematic review and meta-analysis. Radiol. Bras..

[14] Burdach S., Thiel U., Schöniger M., Haase R., Wawer A., Nathrath M., Kabisch H., Urban C., Laws H.J., Dirksen U. (2010). Total body MRI-governed involved compartment irradiation combined with high-dose chemotherapy and stem cell rescue improves long-term survival in Ewing tumor patients with multiple primary bone metastases. Bone Marrow Transpl..

[15] Schäfer J.F., Granata C., von Kalle T., Kyncl M., Littooij A.S., Di Paolo P.L., Sefic Pasic I., Nievelstein R.A.J., Oncology Task Force of the ESPR (2020). Whole-body magnetic resonance imaging in pediatric oncology—Recommendations by the Oncology Task Force of the ESPR. Pediatr. Radiol..

[16] Mhlanga J., Lai H., Alazraki A., Cho S.Y., Nadel H., Pandit-Taskar N., Qi J., Rajderkar D., Voss S., Watal P. (2023). Imaging recommendations in pediatric lymphoma: A COG Diagnostic Imaging Committee/SPR Oncology Committee White Paper. Pediatr. Blood Cancer.

[17] Krohmer S., Sorge I., Krausse A., Kluge R., Bierbach U., Marwede D., Kahn T., Hirsch W. (2010). Whole-body MRI for primary evaluation of malignant disease in children. Eur. J. Radiol..

[18] Klenk C., Gawande R., Uslu L., Khurana A., Qiu D., Quon A., Donig J., Rosenberg J., Luna-Fineman S., Moseley M. (2014). Ionising radiation-free whole-body MRI versus (18)F-fluorodeoxyglucose PET/CT scans for children and young adults with cancer: A prospective, non-randomised, single-centre study. Lancet Oncol..

[19] McCarville M.B., Wu S., Li Y., Kaste S.C., Bishop M., Doubrovin M., Pappo A.S., Krasin M., Hillenbrand C.M. (2025). Comparing Whole-Body Diffusion-weighted MRI to Conventional Imaging: Staging Pediatric Bone and Soft-Tissue Sarcomas. Radiol. Imaging Cancer.

[20] Perkins S.M., Shinohara E.T., DeWees T., Frangoul H. (2014). Outcome for children with metastatic solid tumors over the last four decades. PLoS ONE.

[21] Vali R., Alessio A., Balza R., Borgwardt L., Bar-Sever Z., Czachowski M., Jehanno N., Kurch L., Pandit-Taskar N., Parisi M. (2021). SNMMI Procedure Standard/EANM Practice Guideline on Pediatric (18)F-FDG PET/CT for Oncology 1.0. J. Nucl. Med..

[22] Vaarwerk B., Breunis W.B., Haveman L.M., de Keizer B., Jehanno N., Borgwardt L., van Rijn R.R., van den Berg H., Cohen J.F., van Dalen E.C. (2021). Fluorine-18-fluorodeoxyglucose (FDG) positron emission tomography (PET) computed tomography (CT) for the detection of bone, lung, and lymph node metastases in rhabdomyosarcoma. Cochrane Database Syst. Rev..

[23] Purbhoo K., Vangu M.D. (2022). Normal Variants and Pitfalls of (18)F-FDG PET/CT Imaging in Pediatric Oncology. Front. Nucl. Med..

[24] Pasoglou V., Michoux N., Larbi A., Van Nieuwenhove S., Lecouvet F. (2018). Whole Body MRI and oncology: Recent major advances. Br. J. Radiol..

[25] Mody R.J., Bui C., Hutchinson R.J., Yanik G.A., Castle V.P., Frey K.A., Shulkin B.L. (2010). FDG PET imaging of childhood sarcomas. Pediatr. Blood Cancer.

[26] Kim D.H., Kim S.Y., Lee H.J., Song B.S., Kim D.H., Cho J.B., Lim J.S., Lee J.A. (2011). Assessment of Chemotherapy Response Using FDG-PET in Pediatric Bone Tumors: A Single Institution Experience. Cancer Res. Treat..

[27] Theruvath A.J., Siedek F., Muehe A.M., Garcia-Diaz J., Kirchner J., Martin O., Link M.P., Spunt S., Pribnow A., Rosenberg J. (2020). Therapy Response Assessment of Pediatric Tumors with Whole-Body Diffusion-weighted MRI and FDG PET/MRI. Radiology.

[28] Hempel J.M., Kloeckner R., Krick S., Pinto Dos Santos D., Schadmand-Fischer S., Boeßert P., Bisdas S., Weber M.M., Fottner C., Musholt T.J. (2016). Impact of combined FDG-PET/CT and MRI on the detection of local recurrence and nodal metastases in thyroid cancer. Cancer Imaging.

[29] Rashidi A., Baratto L., Theruvath A.J., Greene E.B., Hawk K.E., Lu R., Link M.P., Spunt S.L., Daldrup-Link H.E. (2022). Diagnostic Accuracy of 2-[(18)F]FDG-PET and whole-body DW-MRI for the detection of bone marrow metastases in children and young adults. Eur. Radiol..

[30] Sepehrizadeh T., Jong I., DeVeer M., Malhotra A. (2021). PET/MRI in paediatric disease. Eur. J. Radiol..

[31] Kleis M., Daldrup-Link H., Matthay K., Goldsby R., Lu Y., Schuster T., Schreck C., Chu P.W., Hawkins R.A., Franc B.L. (2009). Diagnostic value of PET/CT for the staging and restaging of pediatric tumors. Eur. J. Nucl. Med. Mol. Imaging.

[32] Goodman S.Z., Rosenblum J., Tuna I., Levsky J.M., Ricafort R., Taragin B. (2015). Is dedicated chest CT needed in addition to PET/CT for evaluation of pediatric oncology patients?. Clin. Imaging.

[33] Cieszanowski A., Lisowska A., Dabrowska M., Korczynski P., Zukowska M., Grudzinski I.P., Pacho R., Rowinski O., Krenke R. (2016). MR Imaging of Pulmonary Nodules: Detection Rate and Accuracy of Size Estimation in Comparison to Computed Tomography. PLoS ONE.

