# Supplementary material for: Whole-Body MRI Versus 18F-FDG-PET/CT for the Detection of Nodal and Distant Metastases in Pediatric Patients with Solid Tumors
Source: Cancers (Basel). 2026 Aug 24;18(17):2749. doi: 10.3390/cancers18172749 (PMC13564863; doi:10.3390/cancers18172749)
Supplement: Supplementary file 1 [file cancers-18-02749-s001.zip › cancers-4447581-supplementary.pdf]

Table S1. Analysis of the subgroup of patients with small round blue cell tumors (SRBCT): Raw diagnostic performance counts by group and modality.

group	modality	true positive	false positive	false negative	true negative
all SRBCTs (n=14)	^{18}F-FDG-PET/CT	22	8	24	814
	WB-MRI	45	15	1	807
	combination	46	20	0	802
of these: therapy-naïve patients (n=8)	^{18}F-FDG-PET/CT	7	7	3	478
	WB-MRI	10	8	0	477
	combination	10	13	0	472
of these: patients under chemotherapy (n=6)	^{18}F-FDG-PET/CT	15	1	21	336
	WB-MRI	35	7	1	330
	combination	36	7	0	330

Table S2. Analysis of the subgroup of patients with small round blue cell tumors (SRBCT): Results of the sensitivity and specificity analyses assorted by group and modality.

group	modality	sensitivity [% (95% CI[1])]	specificity [% (95% CI[1])]
all SRBCTs (n=14)	^{18}F-FDG-PET/CT	47.8 (32.9–63.1)	99.0 (98.1–99.6)
	WB-MRI	97.8 (88.5–99.9)	98.2 (97.0–99.0)
	combination	100.0 (92.3–100.0)	97.6 (96.3–98.5)
of these: therapy-naïve patients (n=8)	^{18}F-FDG-PET/CT	70.0 (34.8–93.3)	98.6 (97.0–99.4)
	WB-MRI	100.0 (69.2–100.0)	98.4 (96.8–99.3)
	combination	100.0 (69.2–100.0)	97.3 (95.5–98.5)
of these: patients under chemotherapy (n=6)	^{18}F-FDG-PET/CT	41.7 (25.5–59.2)	99.7 (98.4–100.0)
	WB-MRI	97.2 (85.5–99.9)	97.9 (95.8–99.2)
	combination	100.0 (90.3–100.0)	97.9 (95.8–99.2)

[1]CI: confidence interval
